# Evaluation of Diabetes Risk Models Applied to the Prediction of Incident Dysglycaemia in African and European Populations

**DOI:** 10.1111/dom.71285

**Published:** 2026-09-01

**Authors:** Melony Fortuin‐de Smidt, Patrik Wennberg, Hannah Maike Albers, Charles Agyemang, Felix P. Chilunga, Rikard Landberg, Lisa K. Micklesfield, Samuel N. Darko, Julia Otten, Ellis Owusu‐Dabo, Clemens Wittenbecher, Tommy Olsson, Julia H. Goedecke, Ina Danquah

**Affiliations:** ^1^ Department of Public Health and Clinical Medicine Umeå University Umeå Sweden; ^2^ Hertz‐Chair Innovation for Planetary Health, Transdisciplinary Research Area “Technology and Innovation for Sustainable Futures” and Center for Development Research (ZEF) University of Bonn Bonn Germany; ^3^ Department of Public and Occupational Health, Amsterdam Public Health Research Institute, Amsterdam UMC University of Amsterdam Amsterdam the Netherlands; ^4^ Department of Life Sciences, Division of Food and Nutrition Science Chalmers University of Technology Gothenburg Sweden; ^5^ Wallenberg Laboratory, Department of Molecular and Clinical Medicine, Institute of, Medicine University of Gothenburg Sahlgrenska Academy Gothenburg Sweden; ^6^ South African Medical Research Council/WITS Developmental Pathways for Health Research Unit (DPHRU), Department of Paediatrics, Faculty of Health Sciences University of the Witwatersrand Johannesburg South Africa; ^7^ Department of Molecular Medicine Kwame Nkrumah University of Science and Technology Kumasi Ghana; ^8^ Department of Global and International Health Kwame Nkrumah University of Science and Technology Kumasi Ghana; ^9^ SciLifeLab Stockholm Sweden; ^10^ Stellenbosch Institute for Advanced Study (STIAS) Wallenberg Research Centre at Stellenbosch University Stellenbosch South Africa; ^11^ Biomedical Research and Innovation Platform South African Medical Research Council Cape Town South Africa

**Keywords:** calibration, discrimination, ethnicity, incident dysglycaemia, prediction, sub‐Saharan Africa

## Abstract

**Aims:**

We evaluated overall and sex‐specific performance of established diabetes risk models for predicting incident dysglycaemia in African and European populations.

**Materials and Methods:**

We externally evaluated the African Diabetes Risk model, Finnish Diabetes Risk model, Epidemiological Study on the Insulin Resistance Syndrome model and the personal and clinical Framingham models. Evaluation was performed in three prospective cohorts: the Research on Obesity and Diabetes among African Migrants (RODAM‐Pros; migrant and non‐migrant Ghanaians; *n* = 1607), the Middle‐aged Soweto cohort (MASC; urban Black South Africans; *n* = 515) and the Västerbotten Intervention Programme (VIP; Sweden; *n* = 3044). Dysglycaemia was defined according to American Diabetes Association criteria. Discrimination (c‐statistic) and calibration were assessed before and after recalibration.

**Results:**

Dysglycaemia developed in 17.7% of RODAM‐Pros participants, 35.0% of MASC participants and 27.5% of VIP participants, with median follow‐up of 6.7, 6.7 and 9.9 years, respectively. All models demonstrated poor‐to‐modest discrimination (c‐statistic < 0.7). Most models substantially underestimated risk. Recalibration eliminated systematic miscalibration and reduced calibration error to ≤ 2.3% in RODAM‐Pros and VIP and ≤ 3.4% in MASC. Performance varied by sex and cohort.

**Conclusions:**

Existing diabetes risk prediction models can be applied to predict incident dysglycaemia in both Sub‐Saharan African and European populations, but only after recalibration to local risk levels.

## 1 Introduction

Type 2 diabetes accounts for approximately 90% of all diabetes cases and its prevalence is increasing globally, with Sub‐Saharan Africa (SSA) projected to experience the steepest rise, reaching an estimated 142% increase by 2050 [1]. Prediabetes, an intermediate state of hyperglycemia preceding Type 2 diabetes (hereafter referred to as diabetes), is also rising [2]. Importantly, prediabetes is associated with elevated cardiovascular disease and mortality risk even without progression to overt diabetes [3, 4]. Identification of individuals at high risk to develop dysglycaemia (prediabetes and diabetes) is therefore essential for timely, targeted prevention.

Prognostic models support early prevention, clinical decision‐making and resource allocation. However, their performance must be rigorously evaluated in target populations, particularly when developed elsewhere, to avoid reinforcing existing health disparities. This includes assessing applicability, subgroup performance by sex and ethnicity and recalibration performed when necessary, in line with risk‐of‐bias and applicability frameworks [5, 6] and recent guidance on equity‐sensitive use of race in clinical prediction [7].

No prognostic diabetes risk models have been developed in SSA populations or among African migrants [8, 9]. Only one model, the African Diabetes Risk Model (ADRM), was developed using SSA data to detect *undiagnosed* diabetes rather than to predict incident disease [10]. The ADRM [10, 11] and several other diabetes risk models have been validated in SSA settings [11, 12, 13], but mostly for cross‐sectional detection rather than prospective prediction. Consequently, evidence on how well current diabetes risk models predict incident dysglycaemia in SSA populations and globally remains limited.

Population‐ and sex‐specific differences in diabetes pathophysiology and risk factor profiles may further influence model performance. For example, Black Africans typically have higher insulin levels than Europeans at similar insulin sensitivity, despite lower visceral and ectopic fat depots [14]. Associations between traditional risk factors and diabetes also vary by ethnicity and sex [15]. Furthermore, diabetes among individuals without overweight/obesity is more common in SSA countries [16], with a similar increasing trend observed in African Americans [17]. Such differences may lead to systematic miscalibration when prediction models developed in predominantly European populations are applied to SSA cohorts.

We comprehensively evaluated the performance of established invasive (Framingham clinical) and non‐invasive diabetes risk prediction models (ADRM, Finnish Diabetes Risk model (FINDRISK), the Epidemiological Study on the Insulin Resistance Syndrome (DESIR) model and Framingham personal model) for predicting incident dysglycaemia in two SSA and one European cohort and assessed whether performances differed by sex.

## 2 Materials and Methods

This study prospectively evaluated the performance of existing risk prediction models and followed the updated Transparent Reporting of a multivariable prediction model for Individual Prognosis or Diagnosis plus Artificial Intelligence (TRIPOD + AI) checklist [18] (Table S1).

### 2.1 Description of the Cohorts

Three prospective cohorts representing diverse populations were included: (1) the Research on Obesity and Diabetes among African Migrants prospective (RODAM‐Pros) cohort of migrant and non‐migrant Ghanaian adults, (2) the Middle‐aged Soweto cohort (MASC) of urban Black South Africans and (3) the Västerbotten Intervention Programme cohort (VIP) representing a predominantly European population from northern Sweden.

Details are provided in Appendix S1. Briefly, baseline and follow‐up assessments were conducted in RODAM‐Pros (2012–2015; 2019–2022) [19, 20] and MASC (2017–2018; 2023–2025). For VIP, participants with baseline examinations between 2003 and 2011 and a 10‐year follow‐up visit, were included. Ethical approval was obtained from the relevant ethics committees for all cohorts. At baseline and follow‐up, oral glucose tolerance tests (OGTTs) were performed in MASC and VIP, while fasting plasma glucose was measured in RODAM‐Pros.

### 2.2 Study Populations and Outcome Definition

A flow diagram of the inclusions and exclusions for each cohort is presented in Figure 1. Inclusion criteria were: no dysglycaemia at baseline, fasting for at least 8 h and no missing fasting plasma or 2‐h glucose measurements. After exclusions, 1607 RODAM‐Pros, 515 MASC and 3044 VIP participants remained.

**FIGURE 1:**
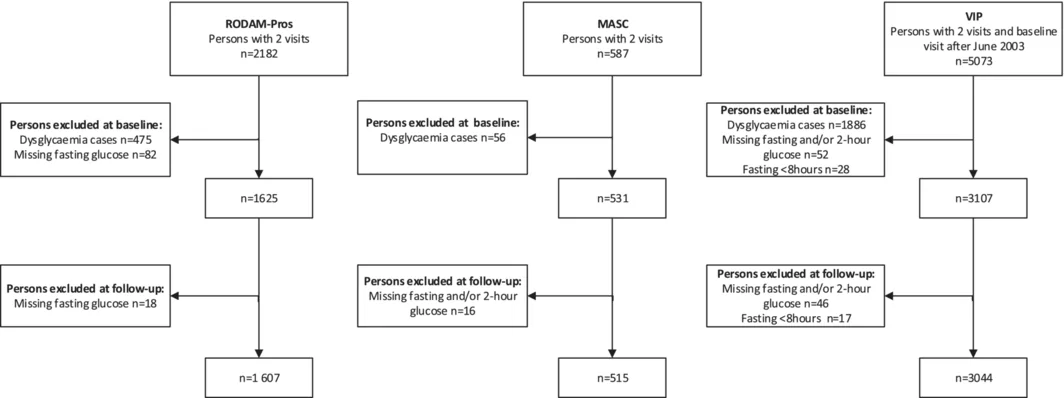
Flow diagram of the study populations included for each cohort: RODAM‐Pros (Research on Obesity and Diabetes among African Migrants Prospective cohort), MASC (Middle‐aged Soweto cohort), VIP (Västerbotten Intervention Programme). Reasons for exclusions were dysglycaemia at baseline, missing glucose measures, and fasting for less than 8 h at baseline and follow‐up.

Dysglycaemia was defined according to American Diabetes Association criteria [21]. In MASC and VIP, dysglycaemia included impaired fasting glucose (IFG), defined as a fasting plasma glucose of 5.6–6.9 mmol/L; impaired glucose tolerance (IGT) defined in MASC as a 2‐h plasma glucose of 7.8–11.0 mmol/L and in VIP as 2‐h capillary glucose of 8.9–12.1 mmol/L; or diabetes. Diabetes was defined as a fasting plasma glucose ≥ 7 mmol/L and/or a 2‐h plasma glucose of ≥ 11.1 mmol/L in MASC, or a 2‐h capillary plasma glucose ≥ 12.2 mmol/L in VIP, and/or the use of anti‐diabetic medication, and/or a validated register diabetes case (only in VIP). In RODAM‐Pros, dysglycaemia was defined as IFG or diabetes, determined by a fasting plasma glucose and/or the use of anti‐diabetic medication [22]. Incident dysglycaemia was defined at the follow‐up visit.

### 2.3 Selection of Prediction Models

We selected five diabetes risk prediction models (Table S2). The ADRM was included as the only model developed in SSA. The FINDRISK model was selected because it is the most commonly validated model in SSA for undiagnosed diabetes [12, 23, 24], and together with the Framingham and the DESIR models, has been validated in eight European countries for incident diabetes [25].

Model selection was further guided by predictor availability across cohorts. Models that included ethnicity were excluded due to the ethnic homogeneity of RODAM‐Pros and MASC, and those incorporating dietary variables were excluded, as these will be assessed in future work. The DESIR model [26] was included because it is sex‐specific. Since invasive models often outperform non‐invasive models but few have been validated in African‐ancestry populations [27], we also included the invasive Framingham clinical model.

### 2.4 Statistical Analysis

Participants with missing data for any model‐specific predictors were excluded, resulting in subsamples of varying sizes (Tables S3–S5). The maximum proportion excluded due to missing was 2.6% in RODAM‐Pros, 0.4% in MASC, and 6.6% in VIP. The standardised mean difference (SMD) was calculated to compare subsamples to the main sample. An absolute SMD ≥ 0.10 was considered indicative of meaningful imbalance [28]. Although sample size was not pre‐specified, each cohort exceeded the rule‐of‐thumb threshold of > 100 events for external validation [29, 30].

Details of model predictors and any modifications are provided in Table S6. In the VIP cohort, HDL‐cholesterol was omitted from the clinical Framingham model evaluation due to > 75% missingness, with a sensitivity analysis conducted in a subset with HDL‐cholesterol available. Additional sensitivity analyses for FINDRISK were performed in RODAM‐Pros and MASC, restricting samples to the original FINDRISK age range (Table S6).

Baseline cohort characteristics were summarised, and sex differences assessed using independent *t*‐tests (parametric continuous variables), the Mann–Whitney U test (non‐parametric continuous variables) and the chi‐squared test (categorical variables).

Predicted probabilities of incident dysglycaemia were calculated using the equations for each risk model (Table S2). The overall performance of the models was assessed using the Brier score, defined as the squared difference between the observed (Y) and predicted probability (*p*) of incident dysglycaemia for each participant (Y‐p)^2^ with lower values indicating better predictive ability. Discrimination was assessed using the concordance statistic (c‐statistic). A c‐statistic ≥ 0.90 is regarded as outstanding, 0.80–0.89 as excellent, 0.70–0.79 as acceptable, 0.60–0.69 as moderate and < 0.60 as poor [31]. In each cohort, the difference in c‐statistics between the models was compared using the DeLong test. To account for multiple comparisons (*n* = 10), the *p*‐values were adjusted using the Benjamini–Hochberg false discovery rate (FDR) procedure.

Calibration, defined as the agreement between predicted and observed risk, was evaluated using: (1) the ratio of the average predicted probability to the average observed probability (E/O ratio), (2) the calibration slope, (3) calibration curves, (4) the integrated calibration index (ICI) and (5) the maximum calibration distance (*E*_max_). An E/O ratio of one indicates no systematic over‐ or underestimation of risk, while a slope of one indicates correct calibration of predictor effects. Confidence intervals for the E/O ratio were estimated using Poisson variance [32]. Calibration curves were used to assess prediction accuracy over the range of predicted probabilities and were summarised using the *E*_max_ [33] and ICI [34]. The ICI, ranging from 0 to 1 (or expressed as a percentage), represents the average absolute difference between the calibration curve and the line of perfect agreement, weighted by the distribution of the predictions. Lower ICI values indicate smaller calibration error and better accuracy. Cohort‐ and sex‐specific model performances were evaluated.

Since the selected models were originally developed for predicting incident or undiagnosed diabetes rather than incident dysglycaemia, some miscalibration was expected. Although these outcomes share risk factors, differences in baseline risk and predictor effects between the development populations and our cohorts can lead to poor agreement between observed and predicted risk, reflected by E/O ratios and calibration slopes deviating from 1. To address this, we recalibrated each model by fitting a logistic regression model with the original linear predictor as the only covariate, yielding updated intercept (baseline risk) and slope (overall predictor strength) estimates using the equation: logit (*p*) = intercept + slope × linear predictor [35, 36]. The recalibrated parameters (Table S7) were then used to adjust predicted probabilities to better reflect dysglycaemia risk distribution within each cohort and by sex.

A clinical utility analysis was conducted, comparing the original and recalibrated models with each other and against the default strategies of intervening in all individuals or in none. Net benefit, which quantifies the benefit of intervening in true‐positive cases against the harm of intervening in false‐positive cases, was plotted across a range of decision thresholds. Clinically relevant thresholds varied between cohorts but generally ranged from 10% to 35%, reflecting differences in baseline risk as well as the relatively low harm associated with preventative interventions in primary care settings such as regular follow‐up and motivational interviewing.

Finally, to evaluate the effect of outcome redefinition, we also assessed model performance for predicting incident diabetes in each cohort in a secondary analysis.

Analyses were performed using Stata 17.0 (STATCorp LP, College Station, Texas, USA) and R (http://www.r‐project.org, R Foundation for Statistical Computing, Vienna, Austria). R Studio (Posit Software, PBC, Boston, Massachusetts, USA) was used to create the calibration plots, to calculate *E*_max_ and ICI (*CalibrationCurves* package v1.0.0) and to perform the decision curve analysis (*d*_curves_ package version 0.5.1). A *p*‐value < 0.05 was considered statistically significant.

## 3 Results

### 3.1 Description of the Selected Models

The five selected prediction models were developed in different populations. The ADRM was developed in SSA Black populations from Tanzania, Senegal and Guinea, whereas the FINDRISK, DESIR and the personal and clinical Framingham models were developed in populations from Europe or European descent living in Finland, France and the USA (Table S2). Age and waist circumference were the most frequently included predictors across models. Smoking was only included in the DESIR model, while systolic blood pressure, fasting plasma glucose, HDL‐cholesterol and triglycerides were only included in the clinical Framingham model. The proportion of participants excluded per model varied from 0.1% to 2.6% in RODAM‐Pros, 0.2%–0.4% in MASC, and 3.3%–6.6% in VIP. Baseline characteristics for each subsample in each cohort are shown in Tables S3–S5. All SMDs comparing each subsample with the main sample were below 0.10, indicating good balance across predictors.

### 3.2 Description of the Cohorts

Baseline characteristics of the cohorts are shown in Table 1. The RODAM‐Pros cohort included a larger proportion of women (66.3%) compared to 46.8% in MASC and 50.5% in VIP. Participants in MASC were older (52.4 ± 6.0 years) than those in RODAM‐Pros (45.5 ± 11.4 years) and VIP (42.9 ± 5.0 years). Obesity prevalence was greatest in MASC (37.7%), intermediate in RODAM‐Pros (21.9%) and lowest in VIP (13.5%).

**TABLE 1:** Baseline characteristics of the RODAM‐Pros cohort, the MASC and the VIP cohort, total and by sex.

	RODAM‐Pros			MASC			VIP		
	Total	Men	Women	Total	Men	Women	Total	Men	Women
*n* (%)	1607	541 (33.7)	1066 (66.3)	515	274 (53.2)	241 (46.8)	3044	1507 (49.5)	1537 (50.5)
Age (years)	45.5 ± 11.4	46.9 ± 12.1	44.8 ± 10.9***	52.4 ± 6.0	52.1 ± 6.2	52.8 ± 5.8	42.9 ± 5.1	42.8 ± 5.0	42.9 ± 5.1
BMI (kg/m^2^)	26.1 ± 5.4	23.9 ± 4.1	27.2 ± 5.5***	28.4 ± 7.1	24.9 ± 5.7	32.4 ± 6.5***	25.3 ± 4.0	26.1 ± 3.6	24.6 ± 4.3***
BMI class *n* (%)									
< 25	739 (46.0)	349 (64.5)	390 (36.6)***	182 (35.3)	150 (54.7)	32 (13.3)***	1629 (53.5)	624 (41.4)	1005 (65.4)***
25–29.9	516 (32.1)	157 (29.0)	359 (33.7)	139 (27.0)	74 (27.0)	65 (27.0)	1047 (34.4)	679 (45.1)	368 (24.0)
≥ 30	351 (21.9)	35 (6.5)	316 (29.7)	194 (37.7)	50 (18.2)	144 (59.8)	367 (12.1)	204 (13.5)	163 (10.6)
Waist (cm)	87.7 ± 12.2	83.9 ± 11.1	89.6 ± 12.3***	92.5 ± 13.6	92.0 ± 14.4	93.0 ± 12.7	87.9 ± 11.8	93.5 ± 10.0	82.4 ± 10.8***
Hip (cm)	98.0 ± 11.3	92.4 ± 8.7	100.9 ± 11.3***	105.8 ± 14.4	98.0 ± 10.9	114.7 ± 12.6***	n/a	n/a	n/a
Systolic blood pressure (mmHg)	126.4 ± 18.1	128.7 ± 18.2	125.2 ± 18***	128.9 ± 19.5	128.7 ± 19.1	129.1 ± 20.0	118.9 ± 14.2	122.3 ± 13.7	115.6 ± 13.8***
Diastolic blood pressure (mmHg)	79.1 ± 11.6	80.9 ± 12.1	78.2 ± 11.2***	87.1 ± 12.2	86.9 ± 12.8	87.4 ± 11.6	74.3 ± 9.9	76.5 ± 9.8	72.1 ± 9.4***
Diabetes family history *n* (%)	288 (17.9)	93 (17.2)	195 (18.3)	126 (24.5)	65 (23.7)	61 (25.3)	536 (18.3)	255 (17.8)	281 (18.8)
Post‐secondary education *n* (%)	68 (4.5)	36 (7.1)	32 (3.2)***	84 (16.3)	60 (21.9)	24 (10.0)**	1129 (37.4)	442 (29.6)	687 (45.1)***
Currently smoking *n* (%)	42 (2.8)	36 (7.2)	6 (0.6)***	161 (31.3)	145 (52.9)	16 (6.6)***	434 (14.4)	202 (13.6)	232 (15.2)
Hypertension *n* (%)	507 (31.6)	180 (33.3)	327 (30.7)	270 (52.5)	135 (49.5)	135 (56.0)	428 (14.1)	250 (16.6)	178 (11.6)***
Fasting glucose (mmol/L)	4.92 (0.56)	5.0 (0.6)	4.9 (0.5)**	4.7 (0.8)	4.7 (0.8)	4.7 (0.7)	5.1 (0.5)	5.1 (0.5)	5.1 (0.6)
2 h glucose (mmol/L)	n/a	n/a	n/a	5.2 ± 1.3	5.1 ± 1.4	5.3 ± 1.1*	6.2 ± 1.1	6.0 ± 1.2	6.4 ± 1.0***
Triglycerides (mmol/L)	0.84 (0.50)	0.9 (0.5)	0.8 (0.5)*	0.8 (0.5)	0.9 (0.6)	0.7 (0.4)**	1.0 (0.6)	1.1 (0.8)	0.9 (0.4)***
HDL‐cholesterol (mmol/L)	1.34 ± 0.38	1.3 ± 0.4	1.4 ± 0.4***	1.4 ± 0.4	1.4 ± 0.4	1.4 ± 0.3	n/a	n/a	n/a

*Note:* Normally distributed continuous variables are described by mean ± standard deviation and non‐normally distributed continuous variables by median (interquartile range). Differences between sexes are assessed using a *t*‐test for normally distributed continuous variables, the Mann–Whitney test for non‐normally distributed continuous variables and the chi‐squared test for categorical variables.

Abbreviations: BMI, body mass index; HDL, high density lipoprotein; MASC, middle‐aged Soweto Cohort; RODAM‐Pros, research on obesity and diabetes among African Migrants Prospective cohort; VIP, Västerbotten Intervention Programme. Hypertension is defined as receiving anti‐hypertensive treatment and/or a systolic and diastolic blood pressure of ≥ 140/90 mmHg.

*p < 0.05.

**p < 0.01.

***p < 0.001.

After a median follow‐up of 6.7 years (interquartile range (IQR) 0.5) in RODAM‐Pros, 6.7 years (IQR 0.9) in MASC and 9.9 years (IQR 0.3) in VIP, the incidence of dysglycaemia was 17.7%, 35.0% and 27.5%, respectively. Incidence of dysglycaemia did not differ significantly between men and women in any cohort.

### 3.3 Model Performance in Each Cohort Before Recalibration

Discrimination was poor to moderate across all models and cohorts with c‐statistics consistently below 0.7 (Table 2). Although the clinical Framingham model achieved the highest discrimination in each cohort, it significantly outperformed only the FINDRISK model in RODAM‐Pros (*p* = 0.022).

**TABLE 2:** Model performance for predicting incident dysglycaemia by cohort before recalibration.

	ADRM	FINDRISK	DESIR	Framingham personal	Framingham clinical
RODAM‐Pros, *n*	1606	1605	1566	1606	1593
C‐statistic (95% CI)	0.66 (0.63–0.70)	0.62 (0.58–0.66)^a^	0.66 (0.62–0.69)^b^	0.65 (0.61–0.68)	0.67 (0.63–0.71)^b^
Brier_original_ (95% CI)	0.169 (0.151–0.187)	0.164 (0.148–0.180)	0.151 (0.135–0.166)	0.161 (0.144–0.177)	0.142 (0.129–0.154)
E/O ratio_original_ (95% CI)	0.09 (0.08–0.10)	0.17 (0.15–0.19)	0.47 (0.42–0.53)	0.23 (0.21–0.26)	0.90 (0.80–1.02)
Slope_original_ (95% CI)	0.58 (0.44–0.72)	0.39 (0.27–0.50)	0.42 (0.32–0.52)	0.62 (0.46–0.78)	0.37 (0.28–0.46)
MASC, *n*	514	513	514	515	514
C‐statistic (95% CI)	0.61 (0.56–0.66)	0.59 (0.54–0.64)	0.59 (0.54–0.64)	0.60 (0.55–0.66)	0.63 (0.58–0.68)
Brier_original_ (95% CI)	0.327 (0.289–0.365)	0.312 (0.275–0.348)	0.277 (0.245–0.308)	0.304 (0.269–0.339)	0.264 (0.234–0.295)
E/O ratio_original_ (95% CI)	0.08 (0.07–0.10)	0.15 (0.13–0.17)	0.39 (0.34–0.46)	0.18 (0.15–0.20)	0.45 (0.39–0.52)
Slope_original_ (95% CI)	0.41 (0.20–0.62)	0.27 (0.12–0.43)	0.24 (0.10–0.38)	0.44 (0.22–0.66)	0.23 (0.14–0.33)
VIP, *n*	2952	2952	2890	2930	2841
C‐statistic (95% CI)	0.59 (0.57–0.62)	0.60 (0.58–0.63)	0.59 (0.57–0.62)	0.59 (0.57–0.61)	0.61 (0.58–0.63)
Brier_original_ (95% CI)	0.266 (0.251–0.281)	0.262 (0.248–0.277)	0.235 (0.221–0.249)	0.254‐ (0.239–0.269)	0.268 (0.259–0.276)
E/O ratio_original_ (95% CI)	0.04 (0.038–0.043)	0.06 (0.055–0.064)	0.25 (0.23–0.26)	0.12 (0.11–0.13)	1.78 (1.66–1.91)
Slope_original_ (95% CI)	0.50 (0.39–0.62)	0.40 (0.32–0.49)	0.29 (0.22–0.36)	0.44 (0.33–0.55)	0.29 (0.22–0.36)

*Note:* The term ‘original’ refers to performance measures prior to recalibration and is used only for measures that are affected by recalibration.

Differences in c‐statistics between models within each cohort are assessed using the DeLong test. *p* values are adjusted for multiple comparisons using the Benjamini–Hochberg false discovery rate procedure.

Abbreviations: ADRM, African diabetes risk model; CI, confidence interval; DESIR, epidemiological study on the insulin resistance syndrome; E/O ratio, expected‐to‐observed probability ratio; FINDRISK, finnish diabetes risk model; MASC, middle‐aged Soweto cohort; RODAM‐Pros, research on obesity and diabetes among African migrants prospective cohort; VIP, Västerbotten intervention programme.

^a^ Indicates a significant difference between the c‐statistic of ADRM and comparator model.

^b^ Indicates a significant difference between the c‐statistic of the FINDRISK and the comparator model.

All models substantially underestimated the risk of incident dysglycaemia, as reflected by E/O ratios well below 1, except for the clinical Framingham model, which overestimated risk by 78% in VIP (Table 2). Among all models, the ADRM showed the greatest underestimation of risk across cohorts (Table 2, Figure S1). Calibration slopes were below 1 for all models and in all cohorts (Table 2), indicating overly extreme predictions.

### 3.4 Model Performance in Each Cohort After Recalibration

Recalibrating the intercept and slope corrected systematic miscalibration across all models. Consequently, E/O ratios and calibration slopes approached 1 in every cohort. Recalibration also improved prediction accuracy across the range of predicted probabilities, reflected by lower ICI and *E*_max_ values (Figure 2).

**FIGURE 2:**
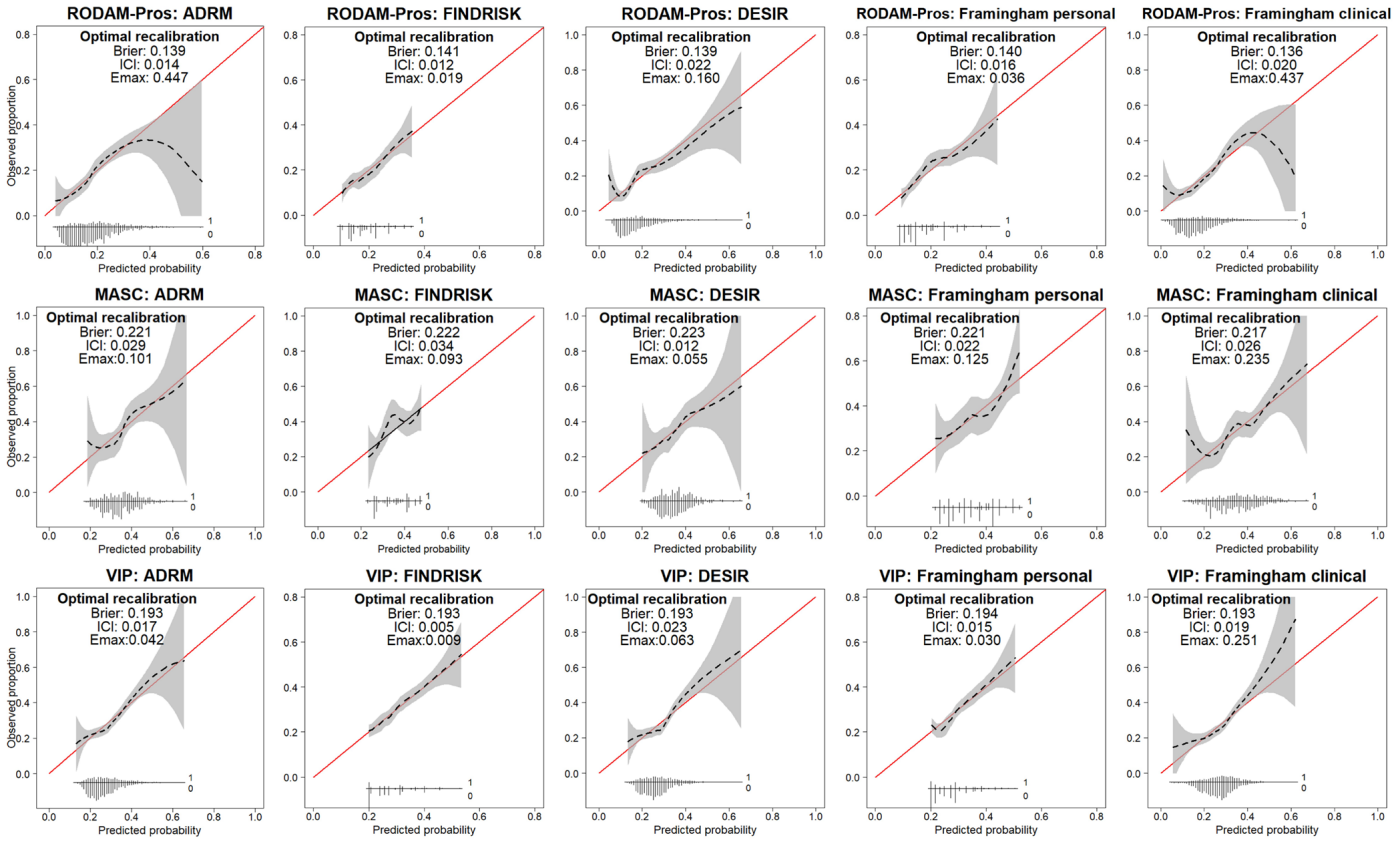
Calibration plots of the ADRM, FINDRISK, DESIR and Framingham models in the Research on Obesity and Diabetes among African Migrants Prospective cohort (RODAM‐Pros), Middle‐Aged Soweto cohort (MASC) and Västerbotten Intervention Programme (VIP) *after recalibration* (slope and intercept adjustment). The red diagonal line represents perfect calibration. The dashed line shows the flexible calibration curve with 95% confidence intervals (grey shading). Bottom vertical lines shows the distribution of predicted probabilities. The Brier score, Integrated Calibration Index (ICI) and maximal calibration error (*E*_max_) are reported.

In RODAM‐Pros, the FINDRISK model was the most accurate over the whole range of predicted risks (ICI 1.2%), followed by the ADRM (ICI 1.4%), while the DESIR model was the least accurate (ICI 2.2%). Similarly in VIP, the FINDRISK model was the most accurate (ICI 0.5%) whereas the DESIR model was least accurate (ICI 2.3%). In contrast, in MASC, the DESIR model had the lowest calibration error (ICI 1.2%) and FINDRISK the highest (ICI 3.4%). The clinical Framingham model tended to underestimate risk in the lower strata of predicted risk in all three cohorts, most prominently in MASC.

### 3.5 Decision Curve Analysis Before and After Recalibration

Across all cohorts, recalibration improved the clinical utility of the prediction models, with recalibrated versions generally providing greater net benefit than the original models (Figure S2). In RODAM‐Pros, the recalibrated models provided greater net benefit than the intervene‐in‐all strategy at threshold probabilities of approximately 10% and above. In VIP, this advantage emerged at threshold probabilities of approximately 22%–25%, while in MASC it was apparent from approximately 30% onwards. Following recalibration, no single model consistently demonstrated superior net benefit across the clinically relevant threshold range, as the decision curves largely overlapped in all cohorts.

### 3.6 Comparative Model Performance in Predicting Incident Diabetes

All models predicting incident diabetes had c‐statistics > 0.7 (Table S8), compared with < 0.7 for incident dysglycaemia (Table 2). In RODAM‐Pros, the Framingham clinical model (0.78, 95% CI 0.74–0.83) had higher discrimination than the other models (DeLong *p* < 0.05), but similar discrimination to the FINDRISK model (0.75, 95% CI 0.71–0.79). In MASC, discrimination was similar across models, whereas in VIP, all models outperformed the Framingham personal model.

Before recalibration, the ADRM, FINDRISK and the Framingham personal model underestimated diabetes risk across all three cohorts (ranging from 74% to 6% underestimation; Table S8), although less so than for incident dysglycaemia risk (Table 2). In contrast, the DESIR and the Framingham clinical models showed outcome‐dependent miscalibration, overestimating diabetes risk (Table S8) but underestimating dysglycaemia risk (Table 2). The Framingham clinical model also showed greater miscalibration and poorer overall performance (higher Brier score) for diabetes than for dysglycaemia.

After recalibration, model accuracy improved across the full range of predicted risks in all cohorts (Figure S3), with less variation in ICI between models than observed for dysglycaemia (Figure 2).

### 3.7 Sex‐Specific Model Performance

Sex‐specific performance measures are shown in Table 3. No model showed superior discrimination within each sex in any cohort, except in RODAM‐Pros men, where the DESIR model had higher discrimination than the FINDRISK model (*p* = 0.035). Before recalibration, overall model performance based on the Brier score was consistently better in women than in men across all cohorts.

**TABLE 3:** Model performance for predicting incident dysglycaemia by cohort and sex before recalibration.

	ADRM	FINDRISK	DESIR	Framingham personal	Framingham clinical	ADRM	FINDRISK	DESIR	Framingham personal	Framingham clinical
RODAM‐Pros	Men					Women				
C‐statistic	0.64 (0.59–0.70)	0.60 (0.54–0.66)	0.66 (0.60–0.71)^a^	0.62 (0.57–0.68)	0.65 (0.59–0.71)	0.67 (0.63–0.72)	0.67 (0.62–0.71)	0.68 (0.63–0.72)	0.66 (0.62–0.71)	0.69 (0.64–0.73)
Brier_orignal_	0.18 (0.15–0.21)	0.18 (0.15–0.21)	0.17 (0.14–0.20)	0.17 (0.14–0.20)	0.15 (0.13–0.18)	0.17 (0.14–0.19)	0.16 (0.14–0.18)	0.14 (0.12–0.16)	0.15 (0.13–0.18)	0.14 (0.12–0.15)
E/O ratio_orignal_	0.08 (0.07–0.10)	0.08 (0.07–0.10)	0.26 (0.21–0.31)	0.18 (0.15–0.22)	0.64 (0.52–0.78)	0.10 (0.09–0.11)	0.22 (0.19–0.25)	0.58 (0.50–0.67)	0.26 (0.22–0.29)	1.05 (0.91–1.22)
Slope_orignal_	0.46 (0.24–0.68)	0.30 (0.07–0.53)	0.47 (0.25–0.70)	0.49 (0.18–0.79)	0.30 (0.15–0.46)	0.66 (0.48–0.84)	0.58 (0.41–0.74)	0.45 (0.33–0.57)	0.71 (0.51–0.91)	0.44 (0.33–0.55)
MASC	Men					Women				
C‐statistic	0.61 (0.54–0.68)	0.64 (0.58–0.71)	0.59 (0.52–0.66)	0.62 (0.55–0.69)	0.65 (0.58–0.72)	0.61 (0.54–0.69)	0.59 (0.51–0.66)	0.60 (0.52–0.68)	0.61 (0.53–0.68)	0.62 (0.54–0.69)
Brier_orignal_	0.35 (0.29–0.40)	0.34 (0.29–0.40)	0.30 (0.25–0.34)	0.33 (0.28–0.38)	0.28 (0.24–0.32)	0.30 (0.25–0.36)	0.27 (0.23–0.32)	0.25 (0.21–0.30)	0.27 (0.23–0.32)	0.25 (0.20–0.29)
E/O ratio_orignal_	0.08 (0.07–0.10)	0.09 (0.07–0.11)	0.34 (0.28–0.42)	0.13 (0.11–0.16)	0.35 (0.28–0.42)	0.09 (0.07–0.11)	0.23 (0.18–0.29)	0.46 (0.37–0.57)	0.24 (0.19–0.30)	0.58 (0.46–0.72)
Slope_orignal_	0.40 (0.13–0.66)	0.44 (0.21–0.67)	0.24 (0.03–0.45)	0.59 (0.27–0.90)	0.26 (0.13–0.39)	0.47 (0.11–0.82)	0.34 (0.07–0.62)	0.24 (0.05–0.44)	0.50 (0.13–0.87)	0.25 (0.09–0.40)
VIP	Men					Women				
C‐statistic	0.62 (0.59–0.65)	0.63 (0.59–0.66)	0.62 (0.58–0.65)	0.61 (0.58–0.64)	0.60 (0.57–0.64)	0.58 (0.55–0.61)	0.58 (0.55–0.61)	0.58 (0.55–0.62)	0.56 (0.53–0.59)	0.60 (0.57–0.64)
Brier_orignal_	0.28 (0.25–0.30)	0.27 (0.25–0.30)	0.24 (0.22–0.26)	0.26 (0.24–0.29)	0.28 (0.27–0.29)	0.26 (0.23–0.28)	0.25 (0.23–0.27)	0.23 (0.21–0.25)	0.24 (0.22–0.26)	0.26 (0.25–0.27)
E/O ratio_orignal_	0.05 (0.04–0.05)	0.05 (0.05–0.06)	0.30 (0.27–0.33)	0.13 (0.12–0.15)	1.80 (1.63–1.98)	0.03 (0.03–0.04)	0.06 (0.06–0.07)	0.19 (0.17–0.21)	0.10 (0.09–0.11)	1.77 (1.60–1.95)
Slope_orignal_	0.67 (0.49–0.85)	0.50 (0.38–0.63)	0.44 (0.31–0.57)	0.57 (0.40–0.73)	0.28 (0.19–0.38)	0.44 (0.27–0.61)	0.31 (0.18–0.43)	0.26 (0.16–0.35)	0.34 (0.18–0.49)	0.28 (0.19–0.37)

*Note:* Performance estimates with the 95% confidence interval in brackets. The term ‘original’ refers to performance measures prior to recalibration and is used only for measures that are affected by recalibration.

Differences in c‐statistics between models within each cohort are assessed using the DeLong test. *p* values are adjusted for multiple comparisons using the Benjamini‐Hochberg false discovery rate procedure.

Abbreviations: ADRM, African diabetes risk model; DESIR, epidemiological study on the insulin resistance syndrome; E/O ratio, expected‐to‐observed probability ratio; FINDRISK, finnish diabetes risk model; MASC, Middle‐aged Soweto cohort; RODAM‐Pros, research on obesity and diabetes among African migrants prospective cohort; VIP, Västerbotten intervention programme.

^a^ Indicates a significant difference between the c‐statistic of the FINDRISK and the comparator model.

After recalibration (Figure S4), sex‐specific differences in overall model performance were attenuated. Model accuracy improved but residual miscalibration remained and differed by cohort and sex. In RODAM‐Pros and VIP, models were generally more accurate in women, as indicated by lower ICI values, whereas in MASC the pattern was inconsistent. In RODAM‐Pros, the clinical Framingham model had the best prediction accuracy within men, but it was least accurate in women, whereas the personal Framingham model performed best in women. In MASC, the DESIR model outperformed the other models in both men and women. In VIP, the personal Framingham model was the only model that was more accurate in men than in women, whereas FINDRISK performed best in women.

### 3.8 Sensitivity Analyses

Restricting the FINDRISK evaluation to participants aged ≤ 64 years in RODAM‐Pros and MASC did not alter model performance (Table S9).

In the VIP subsample with available HDL‐cholesterol (*n* = 664), the clinical Framingham model showed slightly improved discrimination (c‐statistic 0.65, 95% CI 0.60–0.70) compared to the main analysis, and no longer overestimated risk prior to recalibration; instead it underestimated risk (E/O ratio 0.74, 95% CI 0.64–0.85) (Table S9).

## 4 Discussion

### 4.1 Summary of Main Findings

This study provides a comprehensive evaluation of established diabetes risk prediction models applied to predicting *incident dysglycaemia* in populations from Southern Africa (South Africa, MASC), West Africa (Ghana, RODAM‐Pros) and Europe (Sweden, VIP). These models displayed poor‐to‐moderate discrimination and substantial miscalibration prior to recalibration. After simple recalibration of the intercept and slope, systematic miscalibration was eliminated and overall calibration accuracy was markedly improved, although performance varied by cohort and sex. Moreover, recalibration improved the net benefit of all models, but the magnitude and range of clinical utility varied across populations. Together, these findings indicate that existing diabetes risk models can be transported across populations to predict incident dysglycaemia, provided models are adjusted to the local risk profile. However, their ability to distinguish between individuals who will and will not develop dysglycaemia remains limited.

### 4.2 Discrimination of Incident Dysglycaemia

This study is the first to evaluate the ADRM for predicting incident dysglycaemia. Previously, the ADRM was validated for predicting undiagnosed diabetes in rural and urban Black South Africans [10, 11], and in participants from Guinea and Tanzania [10], where it outperformed models developed outside SSA. Contrastingly, our study showed that the ADRM performed comparably to the other models in both SSA cohorts and the European cohort. Unlike earlier validations, which included only non‐invasive models, the current study also evaluated the invasive clinical Framingham model. Models incorporating biochemical predictors such as fasting blood glucose have shown better discrimination [27]; however, when predicting incident dysglycaemia, the clinical Framingham model did not demonstrate superior discrimination compared to most non‐invasive models.

### 4.3 Calibration of the Models

Accurate calibration is essential for clinical decision‐making and communicating individual risk. Since we evaluated existing diabetes risk models to predict incident dysglycaemia, miscalibration was expected. As anticipated, all models substantially underestimated risk across cohorts, except the clinical Framingham model in the Swedish cohort, which overestimated risk.

This overestimation likely reflects the exclusion of HDL‐cholesterol, an inverse predictor of diabetes [37], from the main VIP analysis. When HDL‐cholesterol was included in a subsample, overestimation disappeared.

After slope and intercept adjustment, model accuracy improved markedly across the whole range of predicted risk. However, for some models, specifically the clinical Framingham model, miscalibration persisted within specific risk strata across all three cohorts. Contrastingly, the ADRM predicted risk accurately across most strata in all cohorts, including in Swedish participants. European‐developed models demonstrated the highest accuracy across cohorts: the FINDRISK in RODAM‐Pros and VIP, and the DESIR model in MASC. These results contradict previous evidence that showed poorer performance of European‐developed models in African and Indian populations [38]. However, that study assessed undiagnosed diabetes and focused solely on discrimination.

### 4.4 Clinical Utility of the Models

The clinical utility of the models was strongly influenced by miscalibration, as net benefit improved across all models after recalibration; however, the magnitude of this improvement varied by population. Larger effects were observed in MASC and VIP compared with RODAM‐Pros. Recalibration also broadened the applicable decision range in VIP.

In MASC and VIP, net benefit was observed primarily at higher thresholds compared with RODAM‐Pros. Furthermore, the similarity of the net benefit curves suggests that, despite improved calibration, differences between models remain small, indicating comparable clinical utility.

### 4.5 Comparative Model Performance for Predicting Incident Dysglycaemia Versus Diabetes

While the primary aim was to evaluate model performance for incident dysglycaemia, comparison with incident diabetes provided insight into model transportability across outcomes. As expected, discrimination was higher for incident diabetes, reflecting larger baseline differences between those who develop diabetes and those who remain normoglycaemic. Although most models showed less miscalibration before recalibration than for dysglycaemia, recalibration was still required across all models to improve accuracy. After recalibration, model performance converged for incident diabetes, with no clear advantage of one model over another. In contrast, the greater variability observed for incident dysglycaemia suggests that model choice is more consequential at earlier stages of disease development, with some models better capturing early metabolic risk and demonstrating superior transportability.

### 4.6 The Role of Ethnicity and Sex

Few studies have comprehensively assessed calibration separately in men and women. Our findings show that model accuracy differed by sex and that the pattern of miscalibration was not consistent across cohorts.

For example, the FINDRISK model exhibited relatively high calibration error in Black South African participants but performed well in Ghanaian and Swedish women. One potential explanation relates to the use of WHO‐defined waist circumference categories in FINDRISK [39]. These cut points may not sufficiently capture high dysglycaemia risk in Black South Africans, particularly in women. Indeed, evidence suggests that the recommended waist circumference threshold of 80 cm to predict risk of incident dysglycaemia in women may be too low in Black South African women, and that a higher cut point (91.8 cm) may be more appropriate [40]. Furthermore, the optimal waist circumference thresholds for predicting cardiometabolic risk can vary with the underlying prevalence of obesity [41]. In our study, obesity prevalence was strikingly higher in Black South African women (59.8%) compared with Ghanaian (29.7%) and Swedish women (10.6%), and also higher in Black South African men (18.2%) compared to Ghanaian (6.5%) and Swedish men (13.5%). Models incorporating waist circumference as a continuous variable, instead of fixed threshold waist‐circumference categories, may therefore be preferred in Black South Africans.

Differences in underlying risk factor distributions may explain sex‐specific miscalibration observed in certain models. The clinical Framingham model underestimated risk in lower strata among Ghanaian and Black South African men, a pattern absent in women, other models, or Swedes. The clinical Framingham model may be more prone to underestimate risk in individuals predisposed to dysglycaemia without traditional markers since it includes BMI, waist circumference, and clinical/biochemical variables such as HDL‐cholesterol, triglycerides and fasting plasma glucose [42]. This phenomenon has been documented in SSA populations, where diabetes often occurs in lean individuals. For example, 32% of Ugandan adults with diabetes had BMI < 25 kg/m^2^ [43], and in Ghanaians, the prevalence of diabetes ranged from 13.2% to 55.4% depending on migrant status and rural/urban setting [44]. This lean phenotype is more prevalent in men and associated with lower central obesity and dyslipidaemia, less insulin resistance and greater β‐cell dysfunction [43]. Early‐life undernutrition, linked to β‐cell stress, may contribute to this pattern [45], especially in SSA [46]. Moreover, in Ghanaian men, in whom more than two‐thirds were lean, all models tended to have lower prediction accuracy compared to Black South African and Swedish men, in whom obesity prevalence was higher. Nevertheless, the clinical Framingham model was the most accurate model in Ghanaian men despite the presence of residual miscalibration.

Lastly, another factor to consider is the difference in smoking prevalence in men across cohorts. More than half of Black South African men were smokers, compared with only 7% in Ghanaians and 14.4% in Swedes. This may have contributed to our observed finding that the DESIR model, the only model that included smoking as a predictor in men, together with waist circumference and hypertension, was the most accurate model in Black South African men and the least accurate in both Ghanaian and Swedish men.

Taken together, our findings suggest that sex differences in model performance exist but are context‐specific and reflect underlying risk profiles, such that even in the two SSA cohorts, model performance did not follow similar sex patterns.

### 4.7 Strengths and Limitations

A strength of this study is the use of two well‐characterised SSA cohorts and a large population‐based European cohort, as well as the comprehensive evaluation of discrimination and calibration. Dysglycaemia was assessed using OGTT in two cohorts, improving detection of impaired glucose tolerance [47]. In Ghanaians, dysglycaemia was defined using fasting plasma glucose only, which may have led to under‐detection of isolated impaired glucose tolerance and influenced comparative performance metrics. HDL‐cholesterol was unavailable for most Swedish participants, limiting full evaluation of the clinical FRM, although sensitivity analyses supported our main conclusions. Lastly, follow‐up duration differed between the SSA cohorts (median 6.7 years) and the Swedish cohort (median 9.9 years), which may limit comparability of model performance. However, despite the longer follow‐up in VIP, dysglycaemia incidence was lower than in MASC (27.5% vs. 35.0%), suggesting that underlying population risk, rather than follow‐up duration alone, drives event rates. Differences in follow‐up may still influence the type of cases identified, with shorter follow‐up potentially capturing more rapidly progressing or severe cases, which may contribute to higher discrimination. Consistent with this, a higher proportion of participants in MASC developed Type 2 diabetes (17.0% vs. 6.2% in VIP). Recalibration of the intercept and slope partially mitigated between‐cohort differences by aligning predicted risks with observed event rates and adjusting for differences in predictor effects. However, it does not account for differences in follow‐up structure or outcome ascertainment, which should be considered when interpreting model performance across cohorts.

### 4.8 Research Prospects and Implications for Practice

Our findings highlight the importance of recalibration when applying risk prediction models to new populations, as these simple adjustments markedly improved accuracy. Uncalibrated models should therefore be avoided. Cohort‐specific correction factors could be integrated into app‐based tools, though this may not be feasible in resource‐limited settings. Model performance depended more on included predictors than on the model's population of origin, underscoring the need to select models that capture contextual risk factors. Sex‐specific performance patterns, though inconsistent, suggest potential value in tailored models where risk profiles differ by sex. Context‐specific factors such as diet and physical activity should also be considered in future model development. The clinical Framingham model's underestimation of risk in lean high‐risk men highlights the need to explore predictors relevant to the lean, high‐risk phenotype common in SSA. Finally, risk prediction for incident dysglycaemia remains uncommon but is critical for early prevention. Current evidence shows that interventions in individuals with impaired glucose metabolism can delay diabetes onset but do not fully prevent cardiovascular disease or complications [48]. Identifying those at high risk of developing dysglycaemia, not just diabetes, offers an opportunity for earlier, more effective prevention and could reduce the long‐term burden of hyperglycaemia‐related damage.

## 5 Conclusion

Established diabetes prediction models can be used to predict incident dysglycaemia in both SSA and European populations, but only after recalibration to local baseline risk. Discrimination remains modest, highlighting the need for improved models that capture context‐specific and sex‐specific drivers of dysglycaemia risk.

## Author Contributions

Melony Fortuin‐de Smidt, P.W., H.M.A., C.A., F.P.C., R.L., L.K.M., S.N.D., J.O., Ellis Owusu‐Dabo, C.W., T.O., J.H.G. and I.D. have made substantial contributions to the conception and design, acquisition of data and interpretation of data and reviewed it critically for important intellectual content. Melony Fortuin‐de Smidt has performed the analysis and drafted the manuscript. All authors have approved the final version of the manuscript. Melony Fortuin‐de Smidt is the guarantor of this work, has access to the data and controlled the decision to publish.

## Funding

Funders for the joint project and cohort‐specific funders are acknowledged: This project is supported under the framework of the European Research Area Personalised Medicine Joint Transnational Call (ERA‐PerMed JTC2022), by the South African Medical Research Council (SAMRC) with funds received from the Department of Science and Innovation, Bundesministerium für Bildung und Forschung (BMBF), Germany; Vinnova (Dnr: 2022‐00547) and the Swedish Research Council (Vetenskapsrådet; Dnr: 2022‐01529 for C.W.), Sweden. The handling of participants' sensitive data was enabled by resources provided by the National Academic Infrastructure for Supercomputing in Sweden (NAISS) under project number SENS2023526, partially funded by the Vetenskapsrådet through grant agreement no. 2022‐06725. This work was further supported by the SciLifeLab & Wallenberg National Program for Data‐Driven Life Sciences (grant KAW 2020.0239; C.W.). This study was jointly funded by the South African Medical Research Council (SAMRC) via the South African National Department of Health, the UK Medical Research Council (via the Newton Fund) and the GSK Africa Non‐Communicable Disease Open Lab (grant project number ES/N013891/1) and the South African National Research Foundation (grant number UID:99108). The RODAM‐Pros cohort also received funding from The European Research Council and Federal Ministry of Education and Research. The Västerbotten Intervention Programme is funded by Region Västerbotten. Lastly, Melony Fortuin‐de Smidt was funded by the Matilda Sjölund Diabetes Foundation. The funders were not involved in the design of this study; collection, analysis and interpretation of the data; and did not impose any restrictions regarding the publication of the report.

## Conflicts of Interest

The authors declare no conflicts of interest.

## Supporting information


**Table S1:** TRIPOD‐AI Prediction model validation checklist.
**Appendix S1:** Detailed description of the validation cohorts.
**Table S2:** Description of existing diabetes risk prediction models.
**Table S3:** Describe subsamples created for each model in the RODAM‐Pros cohort.
**Table S4:** Describe subsamples created for each model in the MASC cohort.
**Table S5:** Describe subsamples created for each model in the VIP cohort.
**Table S6:** Description of model variables and cohort‐specific modifications.
**Table S7:** Baseline risk and coefficient strength were updated using the following calibration intercepts and slopes (*β*) obtained from the logistic calibration regression model with the original linear predictor as the only covariate and the observed outcome as the dependent variable (given in logodds).
**Figure S1:** Calibration plots of predicted versus observed risk *before recalibration* for the ADRM, FINDRISK, DESIR and Framingham models in the RODAM‐Pros, MASC and VIP cohorts. The red diagonal line represents perfect calibration. The dashed line shows the flexible calibration curve with 95% confidence intervals (grey shading). Bottom vertical lines shows the distribution of predicted probabilities. The Integrated Calibration Index (ICI) and maximal calibration error (*E*_max_) are reported.
**Figure S2:** Decision curve analysis of the ADRM, FINDRISK, DESIR and Framingham personal and clinical models for predicting incident *dysglycaemia* in the RODAM‐Pros, MASC and VIP cohorts before (dashed lines) and after recalibration (solid lines).
**Table S8:** Model performance for predicting incident diabetes by cohort before recalibration.
**Figure S3:** Calibration plots of the ADRM, FINDRISK, DESIR and Framingham models for predicting incident *diabetes before (grey) and after (blue) recalibration* in the RODAM‐Pros, MASC and VIP cohorts. The red diagonal line represents perfect calibration. Dashed lines show the flexible calibration curve with shaded areas indicating 95% confidence intervals. The Integrated Calibration Index (ICI), maximal calibration error (*E*_max_) and Brier score are reported.
**Figure S4:** Calibration plots by sex for the ADRM, FINDRISK, DESIR and Framingham models in the Research on Obesity and Diabetes among African Migrants Prospective cohort (RODAM‐Pros), Middle‐Aged Soweto cohort (MASC) and Västerbotten Intervention Programme (VIP) *after slope and intercept adjustment* (optimal recalibration). The red diagonal line represents perfect calibration. The dashed line shows the flexible calibration curve with 95% confidence intervals (grey shading). Bottom vertical lines shows the distribution of predicted probabilities. The Brier score, Integrated Calibration Index (ICI) and maximal calibration error (*E*_max_) are reported.
**Table S9:** Sensitivity analysis—Performance measures before and after simple intercept adjustment (recalibration) of the FINDRISK concise model in RODAM‐Pros and MASC *after exclusion of those ≥ 64 years old* and of the Framingham clinical model (in subsample *with HDL‐cholesterol*) in VIP.

## Data Availability

Access to RODAM‐Pros cohort data is based on reasonable requests to the cohort coordinator or principal investigator. MASC data is available on reasonable request from the principal investigators. Access to individual‐level VIP data can be provided for research purposes but is restricted by laws regarding the privacy of research participants and is therefore not made publicly available. Requests for data can be sent to the Section of Biobank Registry Support at Umeå University (contact: info.brs@umu.se).
