# Supplementary material for: Evaluation of Diabetes Risk Models Applied to the Prediction of Incident Dysglycaemia in African and European Populations
Source: Diabetes Obes Metab. 2026 Sep 1;28(11):10814–26. doi: 10.1111/dom.71285 (PMC13646359; doi:10.1111/dom.71285)
Supplement: Supplementary file 1 [file DOM-28-10814-s001.docx]

**Supplementary Materials**

**Evaluation of diabetes risk models applied to the prediction of incident dysglycaemia in African and European populations**

Melony Fortuin-de Smidt^a^ , Patrik Wennberg^a^ , Hannah Maike Albers^b^, Charles Agyemang^c^, Felix P. Chilunga^c^, Rikard Landberg^d,e^, Lisa K. Micklesfield^f^, Samuel Nkansah Darko^g^ ,Julia Otten^a^, Ellis Owusu-Dabo^h^, Clemens Wittenbecher^d,i^, Tommy Olsson^a,j^ , Julia H. Goedecke^a,f,,k^, Ina Danquah^a,b^

^a^ Department of Public Health and Clinical Medicine, Umeå University, Umeå, Sweden

^b^ Hertz-Chair Innovation for Planetary Health, Transdisciplinary Research Area “Technology and Innovation for Sustainable Futures” and Center for Development Research (ZEF), University of Bonn, Bonn, Germany

^c^ Department of Public and Occupational Health, Amsterdam Public Health Research Institute, Amsterdam UMC, University of Amsterdam, Amsterdam, The Netherlands

^d^ Department of Life Sciences, Division of Food and Nutrition Science, Chalmers University of Technology, Gothenburg, Sweden

^e^ Wallenberg Laboratory, Department of Molecular and Clinical Medicine, Institute of

Medicine, University of Gothenburg Sahlgrenska Academy, Gothenburg, Sweden

^f^ South African Medical Research Council/WITS Developmental Pathways for Health Research Unit (DPHRU), Department of Paediatrics, Faculty of Health Sciences, University of the Witwatersrand, Johannesburg, South Africa

^g^ Department of Molecular Medicine, Kwame Nkrumah University of Science and Technology, Kumasi, Ghana

^h^ Department of Global and International Health, Kwame Nkrumah University of Science and Technology, Kumasi, Ghana

^i^ SciLifeLab, Stockholm, Sweden

^j^ Stellenbosch Institute for Advanced Study (STIAS), Wallenberg Research Centre at Stellenbosch University, Stellenbosch 7600, South Africa

^k^ Biomedical Research and Innovation Platform, South African Medical Research Council, Cape Town, South Africa

**Supplementary Table S1:** TRIPOD-AI Prediction model validation checklist

**Supplementary Appendix S1:** Detailed description of the validation cohorts

**Supplementary Table S2:** Description of existing diabetes risk prediction models

**Supplementary Table S3:** Describe subsamples created for each model in the RODAM-Pros cohort

**Supplementary Table S4:** Describe subsamples created for each model in the MASC cohort

**Supplementary Table S5:** Describe subsamples created for each model in the VIP cohort

**Supplementary Table S6**: Description of model variables and cohort-specific modifications

**Supplementary Table S7:** Baseline risk and coefficient strength were updated using the following calibration intercepts and slopes (β) obtained from the logistic calibration regression model with the original linear predictor as the only covariate and the observed outcome as the dependent variable (given in logodds)

**Supplementary Figure S1:** Calibration plots of predicted vs observed risk *before recalibration* for the ADRM, FINDRISK, DESIR and Framingham models in the RODAM-Pros, MASC and VIP cohorts. The red diagonal line represents perfect calibration. The dashed line shows the flexible calibration curve with 95% confidence intervals (grey shading). Bottom vertical lines shows the distribution of predicted probabilities. The Integrated Calibration Index (ICI) and maximal calibration error (Emax) are reported.

**Supplementary Figure S2:** Decision curve analysis of the ADRM, FINDRISK, DESIR, and Framingham personal and clinical models for predicting incident dysglycaemia in the RODAM-Pros, MASC, and VIP cohorts before (dashed lines) and after recalibration (solid lines)

**Supplementary Table S8:** Model performance for predicting incident diabetes by cohort before recalibration

**Supplementary Figure S3:** Calibration plots of the ADRM, FINDRISK, DESIR and Framingham models for predicting incident diabetes *before (grey) and after (blue) recalibration* in the RODAM-Pros, MASC and VIP cohorts. The red diagonal line represents perfect calibration. Dashed lines show the flexible calibration curve with shaded areas indicating 95% confidence intervals. The Integrated Calibration Index (ICI), maximal calibration error (Emax) and Brier score are reported.

**Supplementary Figure S4:** Calibration plots by sex for the ADRM, FINDRISK, DESIR and Framingham models in the Research on Obesity and Diabetes amongst African Migrants Prospective cohort (RODAM-Pros), Middle-Aged Soweto cohort (MASC) and Västerbotten Intervention Programme (VIP) *after slope and intercept adjustment* (optimal recalibration). The red diagonal line represents perfect calibration. The dashed line shows the flexible calibration curve with 95% confidence intervals (grey shading). Bottom vertical lines shows the distribution of predicted probabilities. The Brier score, Integrated Calibration Index (ICI) and maximal calibration error (Emax) are reported.

**Supplementary Table S9:** Sensitivity analysis – Performance measures before and after simple intercept adjustment (recalibration) of the FINDRISK concise model in RODAM-Pros and MASC *after exclusion of those >64 years old* and of the Framingham clinical model (in subsample *with HDL-cholesterol*) in VIP

**Supplementary Table S1:** TRIPOD-AI Prediction model validation checklist^1^

| **Section/Topic** | **Item** | **Checklist Item** | **Page** |
| --- | --- | --- | --- |
| **Title and abstract** | | | |
| Title | 1 | Identify the study as developing and/or validating a multivariable prediction model, the target population, and the outcome to be predicted. | 1 |
| Abstract | 2 | Provide a summary of objectives, study design, setting, participants, sample size, predictors, outcome, statistical analysis, results, and conclusions. | 2,3 |
| **Introduction** | | | |
| Background and objectives | 3a | Explain the medical context (including whether diagnostic or prognostic) and rationale for developing or validating the multivariable prediction model, including references to existing models. | 3-5 |
|  | 3b | Specify the objectives, including whether the study describes the development or validation of the model or both. | 4,5 |
| **Methods** | | | |
| Source of data | 4a | Describe the study design or source of data (e.g., randomized trial, cohort, or registry data), separately for the development and validation data sets, if applicable. | 5,App1 |
|  | 4b | Specify the key study dates, including start of accrual; end of accrual; and, if applicable, end of follow-up. | 5 |
| Participants | 5a | Specify key elements of the study setting (e.g., primary care, secondary care, general population) including number and location of centres. | 5 |
|  | 5b | Describe eligibility criteria for participants. | 6 |
|  | 5c | Give details of treatments received, if relevant. | n/a |
| Outcome | 6a | Clearly define the outcome that is predicted by the prediction model, including how and when assessed. | 6 |
|  | 6b | Report any actions to blind assessment of the outcome to be predicted. | n/a |
| Predictors | 7a | Clearly define all predictors used in developing or validating the multivariable prediction model, including how and when they were measured. | 7, Table S6 |
|  | 7b | Report any actions to blind assessment of predictors for the outcome and other predictors. | n/a |
| Sample size | 8 | Explain how the study size was arrived at. | 7 |
| Missing data | 9 | Describe how missing data were handled (e.g., complete-case analysis, single imputation, multiple imputation) with details of any imputation method. | 7 Table S3-5 |
| Statistical analysis methods | 10c | For validation, describe how the predictions were calculated. | 8, Table S2 |
|  | 10d | Specify all measures used to assess model performance and, if relevant, to compare multiple models. | 8,9 |
|  | 10e | Describe any model updating (e.g., recalibration) arising from the validation, if done. | 9 |
| Risk groups | 11 | Provide details on how risk groups were created, if done. | n/a |
| Development vs. validation | 12 | For validation, identify any differences from the development data in setting, eligibility criteria, outcome, and predictors. | 9, TableS2 |
| **Results** | | | |
| Participants | 13a | Describe the flow of participants through the study, including the number of participants with and without the outcome and, if applicable, a summary of the follow-up time. A diagram may be helpful. | 6, fig1 |
|  | 13b | Describe the characteristics of the participants (basic demographics, clinical features, available predictors), including the number of participants with missing data for predictors and outcome. | 10 Table 1, S3-5 |
|  | 13c | For validation, show a comparison with the development data of the distribution of important variables (demographics, predictors and outcome). | - |
| Model performance | 16 | Report performance measures (with CIs) for the prediction model. | Table 2,3 |
| Model-updating | 17 | If done, report the results from any model updating (i.e., model specification, model performance). | 11, 12 Fig2 |
| **Discussion** | | | |
| Limitations | 18 | Discuss any limitations of the study (such as nonrepresentative sample, few events per predictor, missing data). | 19 |
| Interpretation | 19a | For validation, discuss the results with reference to performance in the development data, and any other validation data. | 15,  16 |
|  | 19b | Give an overall interpretation of the results, considering objectives, limitations, results from similar studies, and other relevant evidence. | 14-19 |
| Implications | 20 | Discuss the potential clinical use of the model and implications for future research. | 20 |
| **Other information** | | | |
| Supplementary information | 21 | Provide information about the availability of supplementary resources, such as study protocol, Web calculator, and data sets. | 22 |
| Funding | 22 | Give the source of funding and the role of the funders for the present study. | 21 |

**Supplementary Appendix S1: Detailed description of the validation cohorts**

The Research on Obesity and Diabetes among African Migrants prospective (RODAM-Pros) ^2^ cohort was designed to investigate the relative contributions of modifiable and non-modifiable risk factors to the development of obesity and diabetes among African migrants and non-migrants. The cohort is based on the earlier cross-sectional RODAM study ^3^, that recruited participants from rural and urban Ghana using multistage random sampling of enumeration areas based on the 2010 census, as well as Ghanaian migrant communities in Amsterdam, the Netherlands. Ghanaians living in Amsterdam were selected through random sampling from the municipal register. Baselines assessments were conducted between 2012 and 2015 and follow-up assessments between 2019 and 2021. The median follow-up was 6.7 years (interquartile range (IQR) 6.4-7.0). Data collection included standardized questionnaires (demography, medical history, socioeconomic status and lifestyle habits), physical examinations and fasting blood sampling.

The Middle-aged Soweto Cohort study (MASC) includes randomly selected men and women who originally participated in the African WITS-INDEPTH Partnerships for Genomic Research (AWI-Gen) Collaborative Centre , a study within the Human Heredity and Health in Africa Consortium ^4^. The AWI-Gen study recruited Black South Africans aged 40-60 years living in Soweto, Johannesburg, South Africa. MASC participants underwent baseline assessments between 2017 and 2018 and follow-up assessments between 2023 and 2025^5^. The median follow-up was 6.7 (IQR 6.2-7.1) years. Data collected included anthropometry, sociodemographic factors, medical history, lifestyle and dietary factors, clinical measurements, and an oral glucose tolerance test (OGTT).

The Västerbotten Intervention Programme (VIP) is a population-based cardiovascular disease prevention initiative that started in 1990 and is embedded in primary care ^6^. Residents of Västerbotten, Sweden, are invited to undergo comprehensive health examinations every 10 years; starting from when they turn 30 (stopped in 1995), thereafter at 40, 50 and 60 years. The median follow-up was 9.9 (IQR 9.8-10.1) years. Assessments included a self-administered questionnaire capturing information on lifestyle and medical history, among other factors. Anthropometric, clinical and biochemical measurements were obtained and an OGTT was also performed.

**Supplementary Table S2:** Description of existing diabetes risk prediction models

|  | African diabetes risk model (ADRM) | Concise Finnish diabetes risk model (FINDRISK) | Epidemiological Study on the Insulin Resistance Syndrome (DESIR) | Framingham personal | Framingham clinical |
| --- | --- | --- | --- | --- | --- |
| Authors, year | Mayige, 2014 ^7^ | Lindström, 2003 ^8^ | Balkau, 2008 ^9^ | Wilson, 2007 ^10^ | Wilson, 2007 ^10^ |
| Country | Tanzania, Senegal, Guinea | Finland | France | USA | USA |
| Data source and year of baseline assessments | Regional cross-sectional surveys: Tanzania - 2006, Senegal - 2010. National cross-sectional survey Guinea - 2009 | National survey conducted in 1987 | Epidemiological Study on the Insulin Resistance syndrome (DESIR) cohort – 1994-1996 | Framingham Offspring Study – mid 1990s | Framingham Offspring Study – mid 1990s |
| Population | Sub-Saharan African Black | European | European | 99% European descent | 99% European descent |
| Follow-up (years) | n/a Diagnostic model | 10 | 9 | 7 | 7 |
| Sample size | 5411 | 4595 | 3817 | 3140 | 3140 |
| Type 2 diabetes cases *n* (%) | 142 (2.7) undiagnosed | 182 (4.1) | 203 (5.3) | 160 (5.1) | 160 (5.1) |
| Outcome ascertainment | FPG (undiagnosed diabetes) | Register-determined antidiabetic medication use | FPG and antidiabetic medication use | FPG and antidiabetic medication use | FPG and antidiabetic medication use |
| Age (mean/ recruitment range) | 39±15 years | 25-64 years | 30-64 years | 54 years (no SD) | 54 years (no SD) |
| Original c-statistic | 0.80 | 0.85 | Men: 0.71, Women: 0.83 | 0.72 | 0.88 |
| Analytical model | Logistic | Logistic | Logistic (Sex-specific) | Logistic | Logistic |
| Handling of missing data | Complete case analysis | Complete case analysis | Not mentioned | Complete case analysis | Complete case analysis |
| Validated in SSA populations | Yes ^11^ | Yes ^11-13^ | No | No | Yes ^13^ |
| Model equation | Predicted probability = $\frac{\exp^{(LP)}}{1+\exp^{(LP)}}$  LP = -11.012+ 0.045 x age (in years) + 0.048 x waist circumference (in cm) + 0.649 (if hypertension, else 0) | Predicted probability = $\frac{\exp^{(LP)}}{1+\exp^{(LP)}}$  LP= -5.514 + 0.628 (if aged 45-54 years) + 0.892 (if aged 55-64 years) + 0.165 (if 25<BMI<30 kg/m2) +1.096 (if BMI>30 kg/m^2^) + 0.857 (if 94<waist circumference (cm)<102 in men, or 80<waist circumference (cm)<88 in women) +1.350 (if waist circumference (cm) >102 in men or > 88 in women) + 0.711 (if using blood pressure medication, else 0) + 2.139 (if positive history of high glucose, else 0) | Predicted probability = $\frac{\exp^{(LP)}}{1+\exp^{(LP)}}$  **Men:**  LP= -10.45 + 0.72 (if current smoker, else 0) + 0.081 x waist circumference (cm) + 0.50 (if hypertension, else 0)  **Women:**  LP = -11.81 + 1.09 (if family history of diabetes, else 0) + 0.095 x waist circumference (cm) + 0.64 (if hypertension, else 0) | Predicted probability = $\frac{\exp^{(LP)}}{1+\exp^{(LP)}}$  LP = -4.499 + log(1.0) if aged <50 years + log(1.54) if aged 50-64 years + log(1.74) if aged >65 years + log(1.25) if male, else 0 + log(1.87) if parental history of diabetes, else 0 + log(1.0) if BMI <25 kg/m^2^ + log(2.35) if BMI 25-29.9 kg/m^2^ + log(6.41) if BMI ≥30 kg/m^2^ | Predicted probability = $\frac{\exp^{(LP)}}{1+\exp^{(LP)}}$  LP = -18.607+ log(0.99)*age(years) + log(0.65) if male, else 0 + log(1.55) if parental history of diabetes, else 0 + log(1.04)*BMI + log(1.01)*SBP + log(0.96)*HDL-cholesterol + log(1.00) *triclycerides + log(1.15)* fasting plasma glucose+ log(1.05)*waist circumference |

Abbreviations: BMI - body mass index, FPG - fasting plasma glucose, HDL - high density lipoprotein, SBP - systolic blood pressure, SD - standard deviation, SSA - Sub-Saharan Africa, LP - linear predictor

**Supplementary Table S3:** Description of model-specific subsamples in the RODAM-Pros cohort (Missing per variable: BMI 1 (0.1%), Waist circumference 1 (0.1%), Smoking 98 (6.1%), Education 98 (6.1%))

|  | ADRM | FINDRISK | DESIR | Framingham personal | Framingham clinical | Full sample |
| --- | --- | --- | --- | --- | --- | --- |
| *n* | 1606 | 1605 | 1566 | 1606 | 1593 | 1607 |
| % excluded due to missing | 0.1 | 0.1 | 2.6 | 0.1 | 0.9 | - |
| Age (years), mean (SD) | 45.5 (11.4) | 45.5 (11.4) | 45.5 (11.3) | 45.5 (11.4) | 45.5 (11.4) | 45.5 (11.4) |
| Women, *n* (%) | 1065/1606 (66.3) | 1064/1605 (66.3) | 1065/1566 (68.0) | 1065/1606 (66.3) | 1054/1593 (66.2) | 1066/1607 (66.3) |
| BMI (kg/m^2^), mean (SD) | 26.1 (5.4) | 26.1 (5.4) | 26.2 (5.4) | 26.1 (5.4) | 26.2 (5.3) | 26.1 (5.4) |
| Waist circumference (cm), mean (SD) | 87.7 (12.2) | 87.7 (12.2) | 87.8 (12.1) | 87.8 (12.1) | 87.7 (12.2) | 87.7 (12.2) |
| Post-secondary education, *n* (%) | 68/1519 (4.5) | 68/1518(4.5) | 68/1512 (4.5) | 68/1519 (4.5) | 68/1507 (4.5) | 68/1520 (4.5) |
| Currently smoking, *n* (%) | 42/1508 (2.8) | 42/1507 (2.8) | 42/1508 (2.8) | 42/1508 (2.8) | 41/1496 (2.7) | 42/1509 (2.8) |
| Diabetes family history, *n* (%) | 287/1606 (17.9) | 286/1605 (17.8) | 281/1566 (17.9) | 287/1606 (17.9) | 282/1593 (17.7) | 288/1607 (17.9) |
| Hypertension, *n* (%) | 507/1606 (31.6) | 507/1605 (31.6) | 493/1566 (31.5) | 507/1606 (31.6) | 505/1593 (31.7) | 507/1607 (31.6) |
| Incident dysglycaemia cases, *n* (%) | 284/1606 (17.7) | 284/1605 (17.7) | 279/1566 (17.8) | 284/1606 (17.7) | 278/1593 (17.5) | 284/1607 (17.7) |

Abbreviations: RODAM-Pros – Research on Obesity and Diabetes amongst African Migrants prospective cohort, ADRM – African Diabetes Risk Model, DESIR – Epidemiological Study on the Insulin Resistance Syndrome, FINDRISK – Finnish Diabetes Risk Model, BMI - body mass index, SD - standard deviation.

The inclusion criterion for each subsample was complete data for all variables included in the respective models: ADRM (age, waist circumference, and hypertension); FINDRISK (age, waist circumference, body mass index, and antihypertensive medication use); DESIR (smoking, hypertension, and waist circumference in men, and family history of diabetes, hypertension, and waist circumference in women); Framingham Personal (age, sex, body mass index, and family history of diabetes); and Framingham Clinical (age, sex, body mass index, waist circumference, systolic blood pressure, family history of diabetes, fasting glucose, HDL cholesterol, and triglycerides).

The standardized mean difference (SMD) for each variable between each subsample and the main sample was calculated. All SMDs were <0.10, indicating no meaningful imbalance.

**Supplementary Table S4:** Description of model-specific subsamples in the MASC cohort (Missing per variable: Waist circumference

1 (0.2%), Hypertension 1 (0.2%))

|  | ADRM | FINDRISK | DESIR | Framingham personal | Framingham clinical | Full sample |
| --- | --- | --- | --- | --- | --- | --- |
| *n* | 514 | 513 | 514 | 515 | 514 | 515 |
| % excluded due to missing | 0.2 | 0.4 | 0.2 | - | 0.2 | - |
| Age (years), mean (SD) | 52.4 (6.0) | 52.4 (6.0) | 52.4 (6.0) | 52.4 (6.0) | 52.4 (6.0) | 52.4 (6.0) |
| Women, *n* (%) | 241/514 (46.8) | 240/513 (46.8) | 241/514 (46.8) | 241/515 (46.8) | 241/514 (46.8) | 241/515 (46.8) |
| BMI (kg/m^2^), mean (SD) | 28.4 (7.2) | 28.4 (7.2) | 28.4 (7.2) | 28.4 (7.1) | 28.4 (7.2) | 28.4 (7.1) |
| Waist circumference (cm), mean (SD) | 92.5 (13.6) | 92.5 (13.6) | 92.5 (13.6) | 92.5 ( 13.6) | 92.5 (13.6) | 92.5 (13.6) |
| Post-secondary education, *n* (%) | 83/514 (16.2) | 83/513 (16.2) | 83/514 (16.2) | 84/515 (16.3) | 83/514 (16.2) | 84/515 (16.3) |
| Currently smoking, *n* (%) | 161/514 (31.3) | 161/513 (31.4) | 161/514 (31.3) | 161/515 (31.3) | 161/514 (31.3) | 161/515 (31.3) |
| Diabetes family history, *n* (%) | 126/514 (24.5) | 125/513 (24.4) | 126/514 (24.5) | 126/515 (24.5) | 126/514 (24.5) | 126/515 (24.5) |
| Hypertension, *n* (%) | 83/514 (16.2) | 269/513 (52.4) | 270/514 (52.5) | 270/514 (52.5) | 270/514 (52.5) | 270/514 (52.5) |
| Incident dysglycaemia, *n* (%) | 180/514 (35.0) | 179/513 (34.9) | 180/514 (35.0) | 180/515 ( 35.0) | 180/514 (35.0) | 180/515 (35.0) |

Abbreviations: MASC – Middle-aged Soweto cohort, ADRM – African Diabetes Risk Model, DESIR – Epidemiological Study on the Insulin Resistance Syndrome, FINDRISK – Finnish Diabetes Risk Model, BMI - body mass index, SD - standard deviation.

The inclusion criterion for each subsample was complete data for all variables included in the respective models: ADRM (age, waist circumference, and hypertension); FINDRISK (age, waist circumference, body mass index, and antihypertensive medication use); DESIR (smoking, hypertension, and waist circumference in men, and family history of diabetes, hypertension, and waist circumference in women); Framingham Personal (age, sex, body mass index, and family history of diabetes); and Framingham Clinical (age, sex, body mass index, waist circumference, systolic blood pressure, family history of diabetes, fasting glucose, HDL cholesterol, and triglycerides).

The standardized mean difference (SMD) for each variable between each subsample (except for the Framingham personal model, which had no missing data) and the main sample was calculated. All SMDs were <0.10, indicating no meaningful imbalance.

**Supplementary Table S5:** Description of model-specific subsamples in the VIP cohort (Missing per variable: BMI 1 (0.03%), Waist circumference 91 (3.0%), Smoking 31 (1%), Diabetes family history 131 (4.3%), Education 24 (0.8%))

|  | ADRM | FINDRISK | DESIR | Framingham personal | Framingham clinical | Full sample |
| --- | --- | --- | --- | --- | --- | --- |
| *n* | 2953 | 2953 | 2891 | 2930 | 2842 | 3044 |
| % excluded due to missing | 3.0 | 3.0 | 5.0 | 3.8 | 6.6 | - |
| Age (years), mean (SD) | 42.8 (5.1) | 42.8 (5.1) | 42.8 (5.1) | 42.9 (5.1) | 42.9 (5.1) | 42.9 (5.1) |
| Women, *n* (%) | 1487/2953 (50.4) | 1487/2953 (50.4) | 1447/2891 (50.1) | 1495/2930 (51.0) | 1446/2842 (50.9) | 1537/3044 (50.5) |
| BMI (kg/m^2^), mean (SD) | 25.3 (3.9) | 25.3 (3.9) | 25.3 (3.9) | 25.3 (4.0) | 25.3 (3.9) | 25.3(4.0) |
| Waist circumference (cm), mean (SD) | 87.9 (11.8) | 87.9 (11.8) | 87.9 (11.8) | 87.8 (11.8) | 87.9 (11.8) | 87.9(11.8) |
| Post-secondary education, *n* (%) | 1101/2931 (37.6) | 1101/2930 (37.6) | 1087/2874 (37.2) | 1106/2911 (38.0) | 1078/2824 (38.2) | 1129/3020 (37.4) |
| Currently smoking, *n* (%) | 421/2924 (14.4) | 421/2924 (14.4) | 410/2885 (14.2) | 414/2905 (14.3) | 401/2818 (14.2) | 434/3013 (14.4) |
| Diabetes family history, *n* (%) | 518/2844 (18.2) | 518/2844 (18.2) | 515/2826 (18.2) | 536/2930 (18.3) | 518/2842 (18.2) | 536/2931 (18.3) |
| Hypertension, *n* (%) | 411/2953 (13.9) | 411/2953 (13.9) | 402/2891 (13.9) | 406/2930 (13.9) | 390/2842 (13.7) | 428/3044 (14.1) |
| Incident dysglycaemia cases, *n* (%) | 807/2953 (27.3) | 807/2953 (27.3) | 785/2891 (27.2) | 803/2930 (27.4) | 774/2842 (27.3) | 837/3044 (27.5) |

Abbreviations: VIP – Västerbotten Intervention Programme, ADRM – African Diabetes Risk Model, DESIR – Epidemiological Study on the Insulin Resistance Syndrome, FINDRISK – Finnish Diabetes Risk Model, BMI - body mass index, SD - standard deviation.

The inclusion criterion for each subsample was complete data for all variables included in the respective models: ADRM (age, waist circumference, and hypertension); FINDRISK (age, waist circumference, body mass index, and antihypertensive medication use); DESIR (smoking, hypertension, and waist circumference in men, and family history of diabetes, hypertension, and waist circumference in women); Framingham Personal (age, sex, body mass index, and family history of diabetes); and Framingham Clinical (age, sex, body mass index, waist circumference, systolic blood pressure, family history of diabetes, fasting glucose, HDL cholesterol, and triglycerides).

The standardized mean difference (SMD) for each variable between each subsample and the main sample was calculated. All SMDs were <0.10, indicating no meaningful imbalance.

**Supplementary Table S6**: Description of model variables and cohort-specific modifications

| **Variables** | **ADRM** | **FINDRISK concise** | **DESIR** | **Framingham personal** | **Framingham clinical** |
| --- | --- | --- | --- | --- | --- |
| **Age (years)** | **Yes**  *continuous* | **Yes**  *categorical: <45, 45-54, 55-64*  *RODAM-Pros and MASC: Persons >64 years old were included in the last category. In a* ***sensitivity analysis*** *persons aged >64 years were excluded (RODAM n=81 and MASC n=5).*  *VIP: no persons in 55-64 year category* | No | **Yes**  *categorical: <50, 50-64, ≥65*  *VIP: no persons in ≥65 years category* | **Yes**  *continuous* |
| **Sex** | No | No | **Yes**^†^ | **Yes** | **Yes** |
| **Diabetes family history** | No | No | **Yes**  *Family history (Yes/No)*  *RODAM: Immediate family*  *MASC: Family history*  *VIP: Parents and siblings* | **Yes**  *Parental history (Yes/No)*  *RODAM: Immediate family*  *MASC: Family history*  *VIP: Parents and siblings* | **Yes**  *Parental history (Yes/No)*  *RODAM: Immediate family*  *MASC: Family history*  *VIP: Parents and siblings* |
| **History of high glucose** | No | **Yes**  *Have you ever been told by a health-care professional that you have diabetes or latent diabetes (Yes/No)*  *RODAM-Pros, MASC and VIP: this variable was excluded from the model because it was not collected.* | No | No | No |
| **Smoking** | No | No | **Yes**  *Current smoker (Yes/No)* | No | No |
| **Body mass index (kg/m^2^)** | No | **Yes**  *categorical: <25, 25-30, >30* | No | **Yes**  *categorical: <25, 25-29.9, ≥30* | **Yes**  *continuous* |
| **Waist circumference (cm)** | **Yes**  *continuous* | **Yes**  *Men categorical: <94, 94 to <102, ≥102*  *Women categorical: <80, 80 to <88, ≥88* | **Yes**  *continuous* | No | **Yes**  *continuous* |
| **Systolic blood pressure, mmHg** | No | No | No | No | **Yes**  *continuous* |
| **Anti-hypertensive medication use** | No | **Yes**  *Have you ever used drugs for high blood pressure? (Yes/No)*  *RODAM-Pros and MASC: Using medication for high blood pressure?*  *VIP: Have you used medication for high blood pressure in the last 14 days?* | No | No | No |
| **Hypertension** | **Yes**  *History of hypertension and/or SBP≥140 mmHg or DBP ≥90 mmHg (Yes/No)*  *RODAM-Pros, MASC, VIP: Mean SBP≥140 mmHg and/or mean DBP≥90 mmHg and/or hypertensive medication use* | No | **Yes**  *Blood pressure ≥140/90 mmHg or on antihypertensive medication (Yes/No)*  *RODAM-Pros, MASC, VIP: Mean SBP≥140 mmHg and/or mean DBP≥90 mmHg and/or hypertensive medication use* | No | No |
| **Fasting glucose (mg/dL)** | No | No | No | No | **Yes**  *continuous* |
| **Triglycerides (mg/dL)** | No | No | No | No | **Yes**  *continuous* |
| **HDL-cholesterol (mg/dL)** | No | No | No | No | **Yes**  *continuous*  *VIP: >75% missing, therefore excluded. A* ***sensitivity analysis*** *was done on a subsample that included HDL- cholesterol.* |
| **Number of variables** | 3 | 5 | 3^ǂ^ | 4 | 9 |

^†^Sex-specific models, ^ǂ^Three variables for men: smoking, waist circumference, hypertension and three variables for women: diabetes family history, waist circumference, hypertension

Abbreviations: SBP – systolic blood pressure, DBP – diastolic blood pressure, HDL – high density lipoprotein

**Supplementary Table S7:** Baseline risk and predictor strength were updated using the following calibration intercepts and slopes obtained from the logistic calibration regression model with the original linear predictor as the only covariate and the observed outcome as the dependent variable (given in logodds)

|  | RODAM-Pros | | MASC | | VIP | |
| --- | --- | --- | --- | --- | --- | --- |
|  | **Intercept** | **slope** | **Intercept** | **slope** | **Intercept** | **slope** |
| ADRM | 1.000284 | 0.5802696 | 0.9571183 | 0.4116646 | 1.3915803 | 0.5018084 |
| FINDRISK | -0.0253656 | 0.3864886 | 0.30922828 | 0.2718872 | 0.83186511 | 0.4017361 |
| DESIR | -0.33113436 | 0.4205723 | -0.071305 | 0.2381814 | -0.07611865 | 0.2921929 |
| Framingham personal | 0.5327284 | 0.6204477 | 0.68176214 | 0.4372139 | 0.6206587 | 0.4397757 |
| Framingham clinical | -0.78221603 | 0.3718778 | -0.01527188 | 0.2330411 | -0.98053359 | 0.2892871 |

Abbreviations: RODAM-Pros – Research on Obesity and Diabetes amongst African Migrants Prospective cohort, MASC- Middle-aged Soweto Cohort, VIP – Västerbotten Intervention Programme, ADRM – African Diabetes Risk Model, FINDRISK – Finnish Diabetes Risk Model, DESIR - Epidemiological Study on the Insulin Resistance Syndrome

**Supplementary Figure S1:** Calibration plots of predicted vs observed risk of incident dysglycaemia *before recalibration* for the ADRM, FINDRISK, DESIR and Framingham models in the RODAM-Pros, MASC and VIP cohorts. The red diagonal line represents perfect calibration. The dashed line shows the flexible calibration curve with 95% confidence intervals (grey shading). Bottom vertical lines shows the distribution of predicted probabilities.The Integrated Calibration Index (ICI) and maximal calibration error (Emax) are reported

**Supplementary Figure S2:** Decision curve analysis of the ADRM, FINDRISK, DESIR, and Framingham personal and clinical models for predicting incident dysglycaemia in the RODAM-Pros, MASC, and VIP cohorts before (dashed lines) and after recalibration (solid lines)

**Supplementary Table S8:** Model performance for predicting incident diabetes by cohort before recalibration

|  | ADRM | FINDRISK | DESIR | Framingham personal | Framingham clinical |
| --- | --- | --- | --- | --- | --- |
| RODAM-Pros, *n* | 1902 | 1901 | 1854 | 1902 | 1892 |
| C-statistic (95%CI) | 0.71 (0.66-0.75) | 0.75 (0.71-0.79)^†^ | 0.74 (0.69-0.78) | 0.71 (0.66-0.75)^¶^ | 0.78 (0.74-0.83)^† § ǂ^ |
| Brier_original_ (95%CI) | 0.063 (0.053-0.074) | 0.058 (0.048-0.067) | 0.064 (0.055-0.072) | 0.061 (0.051-0.071) | 0.113 (0.104-0.122) |
| E/O ratio_original_ (95%CI) | 0.26 (0.22-0.31) | 0.61 (0.51-0.73) | 1.33 (1.11-1.58) | 0.65 (0.54-0.77) | 3.48 (2.92-4.15) |
| Slope_original_ (95%CI) | 0.80 (0.59-1.00) | 0.74 (0.60-0.89) | 0.62 (0.49-0.76) | 0.90 (0.68-1.13) | 0.60 (0.49-0.70) |
| MASC, *n* | 656 | 656 | 656 | 657 | 657 |
| C-statistic (95%CI) | 0.73 (0.68-0.79) | 0.72 (0.66-0.78) | 0.72 (0.67-0.78) | 0.71 (0.65-0.78) | 0.78 (0.72-0.84) |
| Brier_original_ (95%CI) | 0.109 (0.086-0.131) | 0.099(0.080-0.119) | 0.109 (0.093-0.126) | 0.101 (0.082-0.121) | 0.145 (0.125-0.165) |
| E/O ratio_original_ (95%CI) | 0.28 (0.22-0.36) | 0.64 (0.51-0.79) | 1.35 (1.08-1.68) | 0.56 (0.45-0.70) | 2.19 (1.75-2.73) |
| Slope_original_ (95%CI) | 0.90 (0.62-1.19) | 0.61 (0.42-0.80) | 0.54 (0.36-0.72) | 0.95 (0.62-1.27) | 0.44 (0.33-0.55) |
| VIP, *n* | 4570 | 4570 | 4471 | 4578 | 4406 |
| C-statistic (95%CI) | 0.78 (0.75-0.82) | 0.77 (0.74-0.81) | 0.78 (0.74-0.81) | 0.74 (0.70-0.78)^† ¶ §^ | 0.79 (0.75-0.82) ^ǂ^ |
| Brier_original_ (95%CI) | 0.037 (0.032-0.043) | 0.035 (0.030-0.039) | 0.040 (0.036-0.044) | 0.036 (0.031-0.041) | 0.438 (0.428-0.448) |
| E/O ratio_original_ (95%CI) | 0.32 (0.28-0.37) | 0.84 (0.73-0.98) | 2.03 (1.75-2.36) | 0.94 (0.81-1.09) | 15.9 (13.7-18.5) |
| Slope_original_ (95%CI) | 1.30 (1.11-1.49) | 0.74 (0.64-0.84) | 0.82 (0.70-0.94) | 1.09 (0.90-1.27) | 0.65 (0.56-0.74) |

The term ‘original’ refers to performance measures prior to recalibration and is used only for measures that are affected by recalibration. Abbreviations: RODAM-Pros – Research on Obesity and Diabetes amongst African Migrants prospective cohort, MASC – Middle-aged Soweto cohort, VIP – Västerbotten Intervention Programme, ADRM – African Diabetes Risk Model, FINDRISK – Finnish Diabetes Risk Model, DESIR – Epidemiological Study on the Insulin Resistance Syndrome, CI – confidence interval, E/O ratio – expected-to-observed probability ratio.

Differences in c-statistics between models within each cohort are assessed using the DeLong test. *P* values were adjusted for multiple comparisons using the Benjamini-Hochberg false discovery rate procedure.

^†^ indicates a significant difference (*p*<0.05) between ADRM and comparator models.

^¶^ indicates a significant difference (*p*<0.05) between FINDRISK and comparator models.

^§^indicates a significant difference *(p*<0.05) between DESIR and comparator models.

^ǂ^ indicates a significant difference *(p*<0.05) between Framingham personal and comparator models.

**Supplementary Figure S3:** Calibration plots of the ADRM, FINDRISK, DESIR and Framingham models for predicting incident diabetes *before (grey) and after (blue) recalibration* in the RODAM-Pros, MASC and VIP cohorts. The red diagonal line represents perfect calibration. Dashed lines show the flexible calibration curve with shaded areas indicating 95% confidence intervals. The Integrated Calibration Index (ICI), maximal calibration error (Emax) and Brier score are reported.

**Supplementary Figure S4:** Calibration plots by sex for the ADRM, FINDRISK, DESIR and Framingham models in the Research on Obesity and Diabetes amongst African Migrants Prospective cohort (RODAM-Pros), Middle-Aged Soweto cohort (MASC) and Västerbotten Intervention Programme (VIP) *after slope and intercept adjustment* (optimal recalibration). The red diagonal line represents perfect calibration. The dashed line shows the flexible calibration curve with 95% confidence intervals (grey shading). Bottom vertical lines shows the distribution of predicted probabilities. The Brier score, Integrated Calibration Index (ICI) and maximal calibration error (Emax) are reported.

**Supplementary Table S9:** Sensitivity analysis – Performance measures predicting incident dysglycemia before and after recalibration (intercept and slope adjustment) of the FINDRISK concise model in RODAM-Pros and MASC *after exclusion of those >64 years old* and of the Framingham clinical model (in subsample *with HDL-cholesterol*) in VIP

|  | Whole cohort | Men | Women | Whole cohort | Men | Women | Whole cohort | Men | Women |
| --- | --- | --- | --- | --- | --- | --- | --- | --- | --- |
|  | RODAM-Pros FINDRISK | RODAM-Pros FINDRISK | RODAM-Pros FINDRISK | MASC FINDRISK | MASC FINDRISK | MASC FINDRISK | VIP: Framingham clinical | VIP: Framingham clinical | VIP: Framingham clinical |
| *n* | 1526 | 502 | 1022 | 508 | 270 | 238 | 664 | 393 | 271 |
| Dysglycaemia events | 270 | 91 | 179 | 177 | 102 | 75 | 187 | 110 | 77 |
| C-statistic (95%CI) | 0.63(0.59-0.66) | 0.60(0.54-0.67) | 0.67(0.63-0.71) | 0.59(0.54-0.64) | 0.65(0.58-0.72) | 0.59(0.51-0.66 | 0.65(0.60-0.70) | 0.66(0.59-0.72) | 0.66(0.59-0.73) |
| Brier (95%CI) Original | 0.16(0.15-0.18) | 0.18(0.14-0.218) | 0.16(0.14-0.18) | 0.31(0.28-0.35) | 0.35(0.29-0.40) | 0.27(0.22-0.32) | 0.21(0.18-0.23) | 0.200(0.173-0.226) | 0.215(0.178-0.253) |
| Brier (95%CI) Optimal recalibration | 0.14(0.13-0.15) | 0.16 (0.13-0.18) | 0.14(0.12-0.15) | 0.22(0.21-0.23) | 0.22(0.20-0.24) | 0.21(0.19-0.23) | 0.19(0.18-0.21) | 0.19(0.17-0.22) | 0.19(0.17-0.22) |
| Slope (95%CI) | 0.40(0.28-0.52) | 0.31 (0.08-0.55) | 0.57(0.41-0.74) | 0.27 (0.12-0.43) | 0.47(0.23-0.70) | 0.34(0.06-0.62) | 0.34(0.22-0.45) | 0.36(0.20-0.53) | 0.32(0.15-0.49) |
| E/O ratio (95%CI) Original | 0.17(0.15-0.19) | 0.08(0.07-0.10) | 0.21(0.19-0.25) | 0.14(0.13-0.17) | 0.09(0.07-0.11) | 0.23(0.18-0.29) | 0.74(0.64-0.85) | 0.80(0.67-0.97) | 0.66(0.52-0.81) |
| ICI Original | 0.143 | 0.172 | 0.135 | 0.301 | 0.349 | 0.250 | 0.116 | 0.092 | 0.149 |
| ICI Optimal recalibration | 0.011 | 0.031 | 0.008 | 0.033 | 0.056 | 0.040 | 0.022 | 0.033 | 0.040 |
| Emax Original | 0.228 | 0.304 | 0.233 | 0.415 | 0.538 | 0.359 | 0.219 | 0.254 | 0.132 |
| Emax Optimal recalibration | 0.020 | 0.138 | 0.036 | 0.085 | 0.129 | 0.102 | 0.192 | 0.211 | 0.216 |

Abbreviations: RODAM-Pros- Research on Obesity and Diabetes amongst African Migrants Prospective cohort, MASC- Middle-aged Soweto Cohort, VIP – Västerbotten Intervention Programme. FINDRISK – Finnish Diabetes Risk model, CI – confidence interval, E/O ratio – the expected -to-observed probability ratio, ICI – integrated calibration index, Emax – maximum distance between the line of perfect agreement and the calibration curve

**References**

1.	Collins GS, Moons KGM, Dhiman P, et al. TRIPOD+AI statement: updated guidance for reporting clinical prediction models that use regression or machine learning methods. *Bmj.* 2024;385:e078378.

2.	Agyemang C, van der Linden EL, Antwi-Berko D, et al. Cohort profile: Research on Obesity and Diabetes among African Migrants in Europe and Africa Prospective (RODAM-Pros) cohort study. *BMJ Open.* 2022;12(12):e067906.

3.	Agyemang C, Beune E, Meeks K, et al. Rationale and cross-sectional study design of the Research on Obesity and type 2 Diabetes among African Migrants: the RODAM study. *BMJ Open.* 2014;4(3):e004877.

4.	Ali SA, Soo C, Agongo G, et al. Genomic and environmental risk factors for cardiometabolic diseases in Africa: methods used for Phase 1 of the AWI-Gen population cross-sectional study. *Glob Health Action.* 2018;11(sup2):1507133.

5.	Goedecke JH, Nguyen KA, Kufe C, et al. Waist circumference thresholds predicting incident dysglycaemia and type 2 diabetes in Black African men and women. *Diabetes Obes Metab.* 2022;24(5):918-927.

6.	Spath F, Wennberg P, Johansson R, et al. Cohort Profile: The Northern Sweden Health and Disease Study (NSHDS). *Int J Epidemiol.* 2024;54(1).

7.	Mayige M. *Derivation and validation of a simple risk score for undiagnosed diabetes for Tanzania and other African populations.* 2014.

8.	Lindstrom J, Tuomilehto J. The diabetes risk score: a practical tool to predict type 2 diabetes risk. *Diabetes Care.* 2003;26(3):725-731.

9.	Balkau B, Lange C, Fezeu L, et al. Predicting diabetes: clinical, biological, and genetic approaches: data from the Epidemiological Study on the Insulin Resistance Syndrome (DESIR). *Diabetes Care.* 2008;31(10):2056-2061.

10.	Wilson PW, Meigs JB, Sullivan L, Fox CS, Nathan DM, D'Agostino RB, Sr. Prediction of incident diabetes mellitus in middle-aged adults: the Framingham Offspring Study. *Arch Intern Med.* 2007;167(10):1068-1074.

11.	Royce N, Cronje HT, Kengne AP, Kruger HS, Dolman-Macleod RC, Pieters M. HbA1c comparable to fasting glucose in the external validation of the African Diabetes Risk Score and other established risk prediction models in Black South Africans. *BMC Endocr Disord.* 2024;24(1):213.

12.	Omech B, Mwita JC, Tshikuka JG, Tsima B, Nkomazna O, Amone-P'Olak K. Validity of the Finnish Diabetes Risk Score for Detecting Undiagnosed Type 2 Diabetes among General Medical Outpatients in Botswana. *J Diabetes Res.* 2016;2016:4968350.

13.	Mugeni R, Aduwo JY, Briker SM, Hormenu T, Sumner AE, Horlyck-Romanovsky MF. A Review of Diabetes Prediction Equations in African Descent Populations. *Front Endocrinol (Lausanne).* 2019;10:663.
